# Function of gastrin-releasing peptide receptors in ocular itch transmission in the mouse trigeminal sensory system

**DOI:** 10.3389/fnmol.2023.1280024

**Published:** 2023-11-30

**Authors:** Keiko Takanami, Masaya Kuroiwa, Ren Ishikawa, Yuji Imai, Akane Oishi, Midori Hashino, Yasushi Shimoda, Hirotaka Sakamoto, Tsuyoshi Koide

**Affiliations:** ^1^Mouse Genomics Resource Laboratory, National Institute of Genetics (NIG), Mishima, Japan; ^2^Genetics, Research Organization of Information and Systems, Graduate University for Advanced Studies (SOKENDAI), Mishima, Japan; ^3^Department of Environmental Health, Faculty of Human Life and Environmental Sciences, Nara Women’s University, Nara, Japan; ^4^Department of Materials Science and Bioengineering, Nagaoka University of Technology, Nagaoka, Japan; ^5^Technical Section, National Institute of Genetics, Mishima, Japan; ^6^Faculty of Environmental, Life, Natural Science and Technology, Ushimado Marine Institute (UMI), Okayama University, Okayama, Japan; ^7^Department of Biology, Faculty of Environmental, Life, Natural Science, and Technology, Okayama University, Okayama, Japan

**Keywords:** gastrin-releasing peptide receptors, ocular itch, trigeminal sensory system, neuromedin C, histamine, chloroquine

## Abstract

The prevalence of allergic conjunctivitis in itchy eyes has increased constantly worldwide owing to environmental pollution. Currently, anti-allergic and antihistaminic eye drops are used; however, there are many unknown aspects about the neural circuits that transmit itchy eyes. We focused on the gastrin-releasing peptide (GRP) and GRP receptor (GRPR), which are reportedly involved in itch transmission in the spinal somatosensory system, to determine whether the GRP system is involved in itch neurotransmission of the eyes in the trigeminal sensory system. First, the instillation of itch mediators, such as histamine (His) and non-histaminergic itch mediator chloroquine (CQ), exhibited concentration-dependent high levels of eye scratching behavior, with a significant sex differences observed in the case of His. Histological analysis revealed that His and CQ significantly increased the neural activity of GRPR-expressing neurons in the caudal part of the spinal trigeminal nucleus of the medulla oblongata in GRPR transgenic mice. We administered a GRPR antagonist or bombesin-saporin to ablate GRPR-expressing neurons, followed by His or CQ instillation, and observed a decrease in CQ-induced eye-scratching behavior in the toxin experiments. Intracisternal administration of neuromedin C (NMC), a GRPR agonist, resulted in dose-dependent excessive facial scratching behavior, despite the absence of an itch stimulus on the face. To our knowledge, this is the first study to demonstrate that non-histaminergic itchy eyes were transmitted centrally via GRPR-expressing neurons in the trigeminal sensory system, and that NMC in the medulla oblongata evoked facial itching.

## 1 Introduction

Itch is a familiar and important sensation serving as a warning signal in the organism. However, persistent itching often aggravates dermatitis and conjunctivitis due to scratching of the skin and conjunctiva, resulting in a vicious cycle of itching. Such strong and persistent itching is unbearable, impairs the quality of daily life, and has a significant impact on mental health. Allergic conjunctival disease is a typical example of a disease associated with itchy eyes, and its incidence is increasing every year worldwide partly owing to air pollution. The Japanese Ocular Allergy Society reported the prevalence of allergic conjunctivitis to be 48.7% based on an epidemiological survey in 2017 in Japan (Miyazaki et al., 2020). The increase in the number of patients with allergic conjunctival disease due to younger age and aging of the population is also a problem. Seasonal allergic conjunctivitis, including hay fever, refers to the seasonal onset of symptoms, whereas perennial allergic conjunctivitis refers to the perennial onset of symptoms (Takamura et al., 2017; Miyazaki et al., 2020). In Japan, the age distribution of allergic conjunctivitis peaks in teenage years, and women show twice the prevalence of seasonal and perennial allergic conjunctivitis as men (Takamura et al., 2010, 2017; Miyazaki et al., 2020). In China, the number of outpatient visits for allergic conjunctivitis is significantly correlated with NO_2_, O_3_, and temperature, and outpatients are often female (Hong et al., 2016). Dry eyes associated with itchy eyes occur more frequently in women than in men (Sullivan et al., 2017). Currently, anti-allergic eye drops (antihistamines and chemical mediator release inhibitors) are mainly used to treat conjunctivitis; however, no fundamental treatment is available. On the other hand, the neural basis for transmitting itching in the eyes to the brain is unknown.

Two types of neural circuits transmit perception: the spinal sensory system, which transmits perception from the body, and the trigeminal sensory system, which transmits perception from the orofacial area. In the spinal sensory system, gastrin-releasing peptide (GRP) and GRP receptor (GRPR) expressed in the spinal dorsal horn have been reported to specifically transmit acute and chronic itch, based on experiments using mice (Sun and Chen, 2007; Sun et al., 2009; Zhao et al., 2013; Akiyama et al., 2014; Barry et al., 2020; Kiguchi et al., 2020; Kanehisa et al., 2022). In the trigeminal sensory system, we previously reported that GRP is expressed in the trigeminal ganglia and GRPR is specifically expressed in the superficial layer of the caudal part of the spinal trigeminal nucleus (Sp5C) in mice, rats, Suncus, and monkeys (Takanami et al., 2014, 2016, 2022). However, whether GRPR in Sp5C is actually involved in the transmission of facial itching is unclear. Although we previously reported that GRPR-expressing neurons in the Sp5C of rats transmit histamine (His)-induced itchy eyes (Katayama et al., 2022), whether GRPR is involved in the transmission of itchy eyes in the trigeminal sensory system in mice remains unclear.

Itch-specific scratching behavior, an indicator of itching in rodents, has been well analyzed on the rostral back (Kuraishi et al., 1995) and cheek (Shimada and LaMotte, 2008; Akiyama et al., 2010). However, few reports exist on eye-scratching behavior. Nakano et al. (2009) analyzed His-induced eye scratching behavior in mice and found that eye scratching behavior includes an uninterrupted cluster of rapid fore- or hind-limb movements directed to the ocular surface. We also observed His-induced eye scratching behavior in rats and mice (Katayama et al., 2022); however, how non-histaminergic pruritogens act on itchy eyes is unclear.

In this study, we investigated the concentration of pruritogens that elicit eye-scratching behavior using His as a histaminergic pruritogen and chloroquine (CQ) as a non-histaminergic pruritogen, which are commonly used to induce itching in the body area in rodents models. Next, we conducted a histological analysis of the involvement of GRPR in the transmission of itchy eyes through the trigeminal sensory system, behavioral pharmacological analysis of itch using GRPR agonists and antagonists, and analysis of scratching behavior following the induction of cell death in GRPR-expressing neurons using toxins to determine whether GRPR in the trigeminal system is actually involved in the transmission of itchy eyes.

## 2 Materials and methods

### 2.1 Animals

Adult male and female C57BL/6J mice (CLEA Japan, Tokyo, Japan) were used in this study. We used C57BL/6-*Grpr*^*TM*1(*iCre*)^/Bcgen (Grpr-Cre) mice (BIOCYTOGEN, Beijing, China) and Rosa26 loxP-stop-loxP-nlsLacZ (RNZ) mice (Kobayashi et al., 2013; Arakawa et al., 2014; Luo et al., 2016) for the experiments probing the expression site of the *Grpr*. To establish Grpr-Cre mice, frozen spermatozoa of Grpr-Cre (BIOCYTOGEN) were thawed and incubated in CARD FERTIUP^®^ Mouse Sperm Preincubation Medium (Kyudo CO., LTD, Saga, Japan) at 37°C in an atmosphere with 5% CO_2_ for an hour. C57BL/6J female mice were superovulated with intraperitoneal injection of 0.1 mL CARD HyperOva^®^ (Kyudo CO., LTD, Saga, Japan) followed by an intraperitoneal injection of 5 IU human chorionic gonadotropin (Gonatropin, ASKA Animal Health, Tokyo, Japan) 48 h later. Approximately 17 h following gonadotropin injection, the eggs were collected and incubated in human tubal fluid medium (HTF, ARK Resource, Kumamoto, Japan). Immediately after collecting the eggs, preincubated spermatozoa were transferred into a drop of the HTF medium containing eggs, and inseminated eggs were incubated at 37°C. Approximately 5 h later, the fertilized eggs were transferred to a drop of potassium-supplemented simplex optimized medium (KSOM, ARK Resource) and incubated overnight. Two-cell embryos were transferred into the oviducts of pseudopregnant ICR mice (CLEA Japan) that were anesthetized with isoflurane (DS Pharma Animal Health Co., Ltd., Osaka, Japan). Transgenic mice were identified by standard PCR analysis of extracted ear DNA using specific primers. All the mice were maintained on a 12 h light (6:00–18:00)/12 h dark (18:00–6:00) cycle and provided with unlimited access to water and rodent chow. Behavioral analyses were conducted between 13:00 and 18:00. Mice were maintained and all the experiments were performed in accordance with the NIG and Nara Women’s University guidelines; all the procedures were approved by the NIG and Nara Women’s University Committee for Animal Care and Use. All efforts were made to minimize animal suffering and reduce the number of animals used in this study.

### 2.2 Itch behavior

Mature mice aged 10 weeks or older were used in all behavioral analyses. Itch behavior was assessed according to a previously described method (Katayama et al., 2022). All the mice were housed individually for at least 1 week prior to the behavioral analysis. All the mice were habituated singly in an observation cage (an acrylic chamber, 12 cm × 19 cm × 35 cm) more than 2 times before behavioral observation and were acclimated to the observation cage for at least 15 min on the day of the behavioral analysis. Three microliters of saline (Otsuka, Tokyo, Japan) as a control or 50/300 μg histamine (His) (H7125, Sigma, St. Louis, MO) diluted in 3 μL saline or 50/500 μg chloroquine diphosphate salt (CQ) (C6628, Sigma) diluted in 3 μL saline was instilled into the conjunctival sac of the ipsilateral eye or both eyes under anesthesia with isoflurane inhalation. Eye or facial scratching behavior was recorded for 30 min using a SCLABA-Real (Noveltec, Kobe, Japan). The ipsilateral hindfoot always scratched the left or right eye. We counted the pruritogen-evoked hindfoot eye scratching (Supplementary Material: Movie 1), fine and fast forefeet movements (Supplementary Material: Movie 2), and wiping behavior of both the forefeet into the eyes (Supplementary Material: Movie 3) as itch behaviors. Large body movements with both forefeet observed during grooming (Supplementary Material: Movie 4) were excluded from the scratching behavior. For repeated eye stimulation, the analysis days were separated by at least 3 days to 1 week. All the records were made in the absence of the investigators.

### 2.3 Tissue preparation for immunohistochemistry

Vehicle (3 μL saline as a control) or 300 μg His in 3 μL saline or 500 μg CQ in 3 μL saline was instilled into the conjunctival sac of right eye (Immunohistochemistry) or both eyes (Immunofluorescence) for 120 min before fixation. Adult female mice were deeply anesthetized by intraperitoneal injection of pentobarbital sodium (100 mg/kg body weight) (P0776, Tokyo Chemical Industry Co., Ltd., Tokyo, Japan) and transcardially perfused with physiological saline followed by 4% paraformaldehyde in 0.1 M phosphate buffer (PB). Brainstem and upper cervical spinal cords were removed, immersed in the same fixative for 1 h at room temperature, and then immersed in 25% sucrose in 0.1 M PB for 48 h at 4°C. Then, tissues were embedded in OCT compound and quickly frozen and cut into 30 μm-thick sections on a cryostat (CM3050 S; Leica, Nussloch, Germany). These sections were then washed several times (5 min/wash) with phosphate-buffered saline (PBS).

### 2.4 Immunohistochemistry and immunofluorescence

Immunohistochemistry was conducted according to our previously described methods (Takanami et al., 2021). Sections were first incubated with 1% H_2_O_2_ in absolute methanol for 20 min to eliminate endogenous peroxidase activity. Sections were then rinsed with PBS three times (5 min/rinse). After blocking non-specific binding with 1% normal goat serum and 1% BSA in PBS containing 0.3% Triton X-100 for 30 min at room temperature, sections were incubated with the primary rabbit antiserum raised against human c-Fos (1:10,000; ab190289, Abcam, Cambridge, UK; RRID:AB_2737414) for 24 h at room temperature. The sections were then incubated for 2 h at room temperature with biotinylated-goat anti-rabbit IgG antibody (1:1,000; BA-1000, Vector laboratories, Newark, CA). Immunolabeling was detected using a VECTASTAIN Elite ABC Standard Kit (PK-6100, Vector laboratories) followed by diaminobenzidine development (049-22831, Wako DAB Tablet, Osaka, Japan). Immunoreacted sections were captured and analyzed using a Keyence BZ-X710 (Keyence, Osaka, Japan). Using 3-5 cross sections per mice of the caudal medulla, both sides of the caudal part of the spinal trigeminal nucleus (Sp5C) with c-Fos-immunopositive neurons were photographed. The number of c-Fos positive cells in the Sp5C was analyzed using BZ-X Analyzer (Keyence).

Immunofluorescence for c-Fos and beta-galactosidase (β-gal), an indicator of *Grpr* expression was conducted according to our previously described methods (Takanami et al., 2022). Non-specific binding components were blocked with 1% normal donkey serum and 1% bovine serum albumin in PBS containing 0.3% Triton X-100 for 1 h at room temperature. Sections were incubated with the primary rabbit antiserum raised against human c-Fos (1:2,000; Abcam) and the anti-β-gal mouse antibody (1:500; Z378A, Promega, Madison, WI; RRID:AB_2313752) for the detection of LacZ expression for overnight at 4*^o^*C. The sections were then incubated for 2 h at room temperature with Alexa Fluor 488-linked anti-mouse IgG raised in donkeys (1:1,000; Molecular Probes, Eugene, OR) and Alexa Fluor 555-kinked anti-rabbit IgG raised in donkeys (1:1,000; Molecular Probes). Immunoreacted sections were imaged by using a confocal laser scanning microscope (FV1000, Olympus, Tokyo, Japan; Nikon C2^+^, Nikon, Tokyo, Japan). Images were captured and saved as TIFF format. Analyses of c-Fos and β-gal immunoreactivity in the Sp5C were performed on brainstem cross-sections. We counted the number of c-Fos^+^/β-gal^+^/neurons in the lateral and ventral areas of the superficial layers of the Sp5C. Double^+^ neurons were also counted. The cell counts for each signal were obtained with at least four sections from each mouse.

### 2.5 Intracisternal injection

The intracisternal injection was performed according to previously reported methods reported (Ueda et al., 1979; Fujii et al., 2019). A stainless-steel needle (27G × 3/4e needle, NN-2719S, TERUMO, Tokyo, Japan) with its tip bent at 40° was inserted along the occipital bone of the mouse between the occipital bone and atlas into the cisterna magna under anesthesia with isoflurane. Based on the confirmation of the spread of the dye to the caudal part of the brainstem by 1% methylene blue injection and behavioral observations after injection, a total volume of 5 μL injection with a 3.1–3.3 mm needle tip was performed in 18.5–22.0 g female mice. The fur on the rostral back was shaved for intracisternal injection at least three days prior to administration. For the GRPR agonist injections, either vehicle, artificial cerebrospinal fluid (ACSF) (3525, Tocris Bioscience, Bristol, UK), or the GRPR agonist, [Ser] neuromedin C (NMC) (0.001–1 nmol; SCRUM, Tokyo, Japan) was administered (5 μL), referring to previous reports of intrathecal administration. For GRPR antagonist injections, vehicle, ACSF, or the GRPR antagonist RC-3095 (0.1–1 nmol; R9653; Sigma-Aldrich, St. Louis, MO) was administered (5 μL), followed 10 min later by 3 μL of saline or 300 μg His or 500 μg CQ instillation into the eyes. The GRPR agonist and antagonist dosing concentrations were based on previous reports on intrathecal administration (Sun and Chen, 2007; Sukhtankar and Ko, 2013; Akiyama et al., 2014; Kiguchi et al., 2020).

### 2.6 Toxin treatment

Either control, 500 ng blank-saporin or 500 ng bombesin-saporin *(*KIT-40, Advanced Targeting Systems, Carlsbad, CA*)* was intracisternally administered (5 μL volume) 2 weeks prior to the behavioral experiments as reported (Sun et al., 2009).

### 2.7 Open field test

Open field test was conducted according to our previously described methods (Horii et al., 2017). Behavior was observed for 10 min with a mouse in a square open field arena (60 × 60 cm) made of white polyvinylchloride plastic board with walls 40 cm high. Each mouse was gently picked up by their tails using large tweezers covered with silicon tubing to alleviate pain, and placed gently in the same corner of the open field. During the 10 min trial, their behavior was recorded continually using a video camera placed over the center of the arena, and several parameters, including the distance traveled and time spent in the center of the arena, were determined using a video tracking system (Image OF; Ohara Co. Ltd., Tokyo, Japan) using National Institutes of Health ImageJ software.

### 2.8 Statistical analysis

All the data are expressed as the mean ± standard error of the mean. Statistical analyses of the effects of substance concentration on scratching behavior in each sex were performed using one-way analysis of variance (ANOVA) followed by *post-hoc* Dunnett’s test. Mann–Whitney *U* test was used to examine sex differences in scratching behavior. Two-way ANOVA and *post-hoc* Bonferroni correction for the multiple comparisons were used for analysis of ipsilateral and contralateral side c-Fos expression by saline or CQ instillation. For unpaired-2 group statistics, Student’s or Welch’s *t*-test or Mann–Whitney *U* test was used to analyze the His- or CQ-induced itch behavior or immunofluorescence for the itch mediators. One-way ANOVA and *post-hoc* Dunnett’s multiple comparison test were used to compared the NMC-evoked facial itch behavior. Repeated one-way ANOVA combined with *post-hoc* Durbin-Conover test with Bonferroni correction for the multiple comparisons was used for analysis of the effects of GRPR antagonist on the itch behavior induced by His or CQ. One-way ANOVA and *post-hoc* Tukey’s multiple comparison test were used to compared the effect of bombesin-saporin on the itch behavior induced by His or CQ. Differences were considered statistically significant when the *P-*value was <0.05. All the data were analyzed using SPSS Statistics version 27 (IBM, Chicago, IL, USA) or the open-source statistical software, jamovi version 2.3.28 (The jamovi project).^1^ Graphs were generated using GraphPad Prism 9 software (GraphPad Software, San Diego, CA, USA).

## 3 Results

### 3.1 Dose-dependent increase and sex differences in itchy-eyes behavior

**FIGURE 1:**
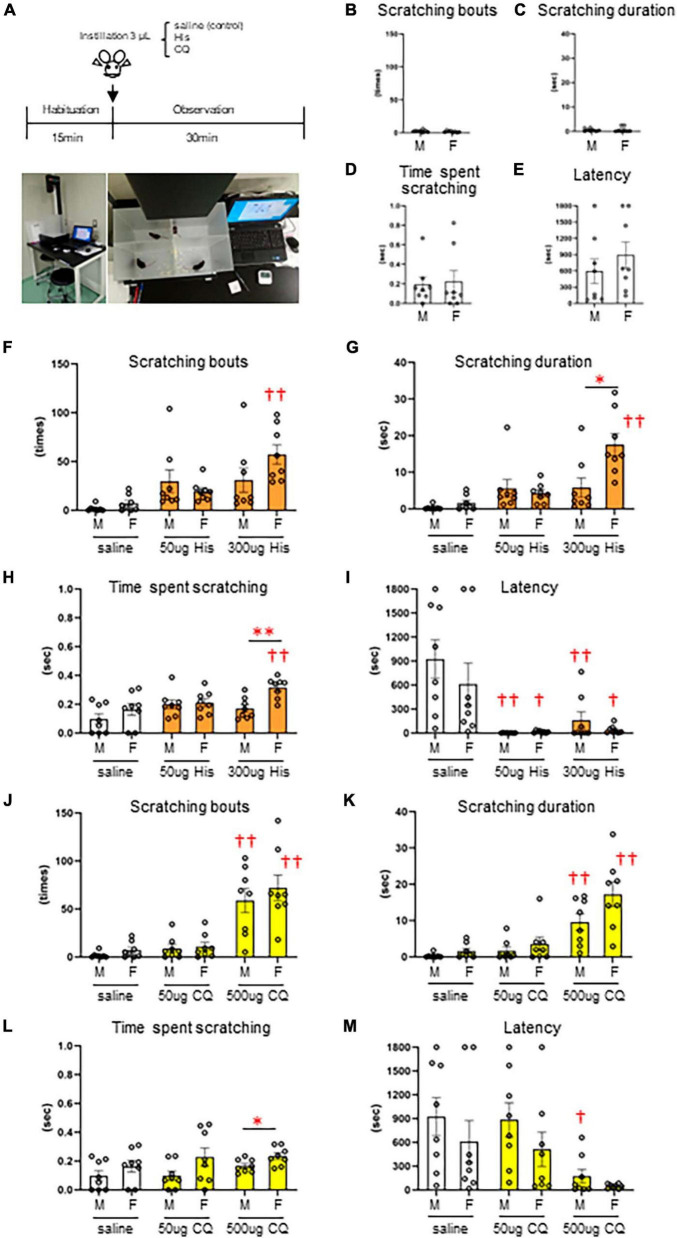
Dose-dependent increase and sex differences in itchy-eyes behavior induced by histamine (His) and chloroquine (CQ). **(A)** Time course. **(B–E)** Spontaneous itch behavior. **(F–I)** His-induced itch behavior. **(F)** Female mice showed higher scratching bouts evoked by 300 μg His than saline (control) instillation (*post-hoc* Dunnett’s test: *P* < 0.01). **(G)** Female mice showed higher scratching duration evoked by 300 μg His than saline instillation (*post-hoc* Dunnett’s test: *P* < 0.01) and male mice (Mann–Whitney *U* test: *P* < 0.05). **(H)** Female mice showed higher time per scratching bout at 300 μg His than saline instillation (*post-hoc* Dunnett’s test: *P* < 0.01) and male mice (Mann–Whitney *U* test: *P* < 0.05). **(I)** Males and females showed a significant decrease in latency after His eye instillation (*post-hoc* Dunnett’s test: 50 μg or 300 μg in males; *P* < 0.01, 50 μg or 300 μg in females; *P* < 0.05). **(J–M)** CQ-induced itch behavior. Male and female mice showed higher scratching bouts **(J)** and scratching duration **(K)** evoked by 500 μg CQ than saline (control) instillation (*post-hoc* Dunnett’s test: *P* < 0.01). **(L)** Female mice showed higher time per scratching bout evoked by 500 μg CQ than male mice (Mann–Whitney *U* test, *P* < 0.05). **(M)** Male mice showed a significant decrease in latency after 500 μg CQ eye instillation (*post-hoc* Dunnett’s test: *P* < 0.05). *: male (*n* = 8) vs. female (*n* = 8). †: saline vs. 50 μg His or 300 μg His, saline vs. 50 μg CQ or 500 μg CQ. *P < 0.05; **P < 0.01; †*P* < 0.05; †⁣†*P* < 0.01.

Since there are very few reports on the concentration of pruritogens that elicit eye itch in mice, we first performed a behavioral analysis of itchy eyes in male (*n* = 8) and female (*n* = 8) mice (Figure 1A). Limited eye-scratching behavior was observed in the absence of stimulation in both sexes (Figures 1B–E): spontaneous). In His instillation into both eyes, there was no difference in the number of scratches [*F*(2, 21) = 2.813, *P* = 0.083], the scratching duration [*F*(2, 21) = 2.247, *P* = 0.131], or the time per scratching bout [*F*(2, 21) = 2.828, *P* = 0.082] for low (50 μg) or high (300 μg) concentrations of His compared to control (saline instillation) in male mice (Figures 1F–I). On the other hand, high concentration of 300 μg His significantly increased the number of scratches [*F*(2, 21) = 17.010, *P* < 0.001, *post-hoc* Dunnett’s test: †⁣†*P* < 0.001] (Figure 1F), the scratching duration [*F*(2, 21) = 20.720, *P* < 0.001, *post-hoc* Dunnett’s test: †⁣†*P* < 0.001] (Figure 1G), and the time per scratching bout [*F*(2, 21) = 5.794, *P* = 0.010, *post-hoc* Dunnett’s test: †⁣†*P* < 0.01, *P* = 0.006] (Figure 1H) compared to the control (saline instillation) in female mice. Both males and females showed a significant decrease in latency until first scratching after His eye instillation compared to the control [*F*(2, 21) = 10.670, *P* < 0.001, *post-hoc* Dunnett’s test: 50 μg male; †⁣†*P* < 0.001, 300 μg male; †⁣†*P* < 0.01, *P* = 0.003], [*F*(2, 21) = 4.858, *P* = 0.018, *post-hoc* Dunnett’s test: 50 μg female; †*P* < 0.05, *P* = 0.022, 300 μg female; †*P* < 0.05, *P* = 0.028] (Figure 1I). At high concentrations of His instillation, a significant sex difference was observed in the scratching duration (Mann–Whitney *U* test; *P < 0.05, *P* = 0.01) (Figure 1G) and time per scratching bout (Mann–Whitney *U* test: **P < 0.01, *P* = 0.005) (Figure 1H). In CQ instillation into both eyes, both males and females showed a significant increase in the number of scratches [males: *F*(2, 21) = 16.574, *P* < 0.001, *post-hoc* Dunnett’s test: †⁣†*P* < 0.001, females: *F*(2, 21) = 18.869, *P* < 0.001, post-hoc Dunnett’s test: †⁣†*P* < 0.001] (Figure 1J) and the scratching duration in males and females [males: *F*(2, 21) = 12.441, *P* < 0.001, *post-hoc* Dunnett’s test: †⁣†*P* < 0.001, females: *F*(2, 21) = 14.088, *P* < 0.001, post-hoc Dunnett’s test: †⁣†*P* < 0.001] (Figure 1K) at the higher concentration of 500 μg CQ. Male mice showed a significant decrease in latency until the first scratching after 500 μg CQ eye instillation [*F*(2, 21) = 4.798, *P* = 0.019, *post-hoc* Dunnett’s test: †*P* < 0.05, *P* = 0.022] (Figure 1M). At high concentration of CQ, a significant sex difference was observed in the time per scratching bout (Mann–Whitney *U* test: *P < 0.05, *P* = 0.028) (Figure 1L). His and CQ instillation into both eyes induced several behavior including hindfoot eye scratching, fine and fast forefeet movements, and facial wiping behavior of both the forefeet (Supplementary Material: Movie 1–3). Thus, we found that both His and CQ significantly increased eye scratching behavior at high concentrations, and female dominant sex differences in scratching were observed. Therefore, we applied eye instillation of high concentrations of His and CQ and used female mice in subsequent experiments.

### 3.2 Itchy eyes induced the activation of the lateral part of the Sp5C neurons

**FIGURE 2:**
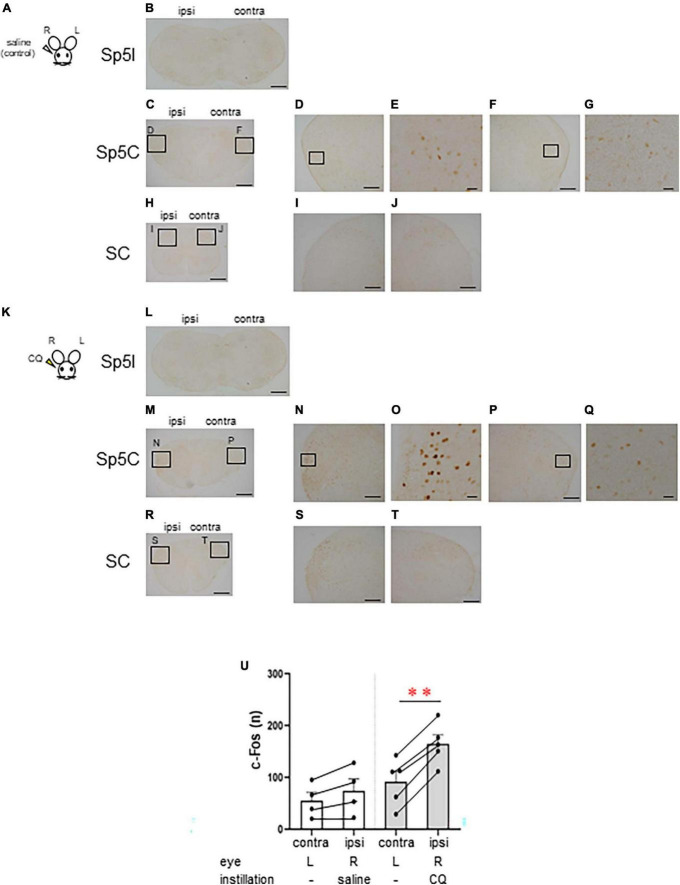
Chloroquine (CQ) instillation induced the activation of the lateral part of the caudal part of the spinal trigeminal nucleus (Sp5C) neurons in the medulla. Representative c-Fos immunostaining in the brainstem and cervical spinal cord after saline **(A–J)** or 500 μg CQ **(K–T)** instillation into the right eye. **(B, L)** The interpolar part of the spinal trigeminal nucleus (Sp5I). **(C–G, M–Q)** Sp5C. **(H–J, R–T)** Upper cervical spinal cord. **(D, F)**, **(I, J)**, **(N, P)**, and **(S, T)** are enlargements of the boxed areas in panels **(C, H, M, R)**, respectively. **(E, G, O, Q)** are enlargements of the boxed areas in panels **(D, F, N, P)**, respectively. Bars = 500 μm **(B, C, H, L, M, R)**; 200 μm **(D, F, I, J, N, P, S, T)**; 50 μm **(E, G, O, Q)**. (U) c-Fos-expressing neurons in the ipsilateral side of the Sp5C increased significantly more than the contralateral side in CQ-treated mice (*post-hoc* Bonferroni correction: *P* < 0.01). Saline instillation group (*n* = 4), 500 μg CQ instillation (*n* = 5). ipsi, ipsilateral side; contra, contralateral side.

Next, we examined the area of neural activation from the brainstem to the upper cervical spinal cord when itching stimuli were applied to the eye. We compared the neural activity in these area between the group that received saline instillation in the right eye as a control (*n* = 4, female) (Figures 2A–J) and the group that received 500 μg CQ eye instillation in the right eye (*n* = 5, female) (Figures 2K–T). Both groups were instilled in the right eye only and not in the left eye. c-Fos-positive neurons were rarely observed in the interpolar part of the spinal trigeminal nucleus (Sp5I) in both the saline (Figure 2B) and CQ (Figure 2L) instillation groups. c-Fos-positive neurons were densely observed on the ipsilateral side of the Sp5C (Figures 2M–Q) and the upper cervical spinal cord (Figures 2R–T) in the CQ instillation group compared to that in the control group (Figures 2C–J). There was a difference in the pattern of c-Fos expression between the saline- and CQ-instillation groups [*F*(1, 7) = 31.501, *P* < 0.001]. Although the number of c-Fos-expressing neurons in the Sp5C on the contralateral and ipsilateral sides did not differ in the saline-treated group, the number of c-Fos-expressing neurons on the ipsilateral side of the Sp5C increased significantly compared to those on the contralateral side in the CQ-treated group (*post-hoc* Bonferroni correction: **P < 0.001) (Figure 2U).

### 3.3 Histaminergic and non-histaminergic pruritogen-induced itchy eye stimulation increased the neural activity of the GRPR-expressing neurons in the Sp5C

**FIGURE 3:**
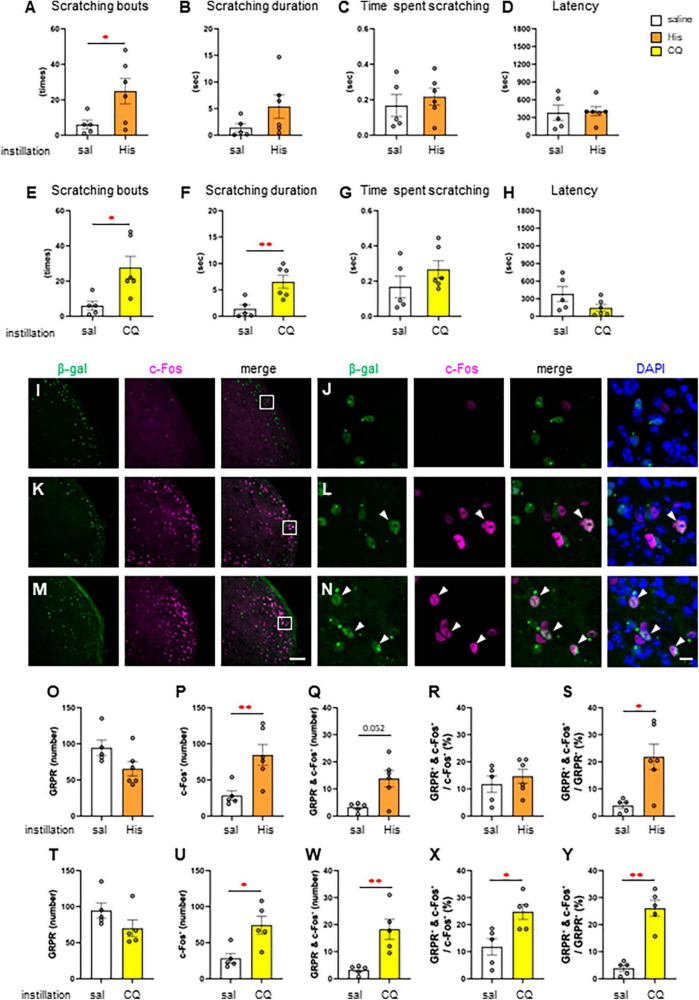
Histamine (His) or chloroquine (CQ)-induced itchy eye increased the neural activity of gastrin-releasing peptide receptor (GRPR)-expressing neurons in the caudal part of the spinal trigeminal nucleus (Sp5C). **(A–D)** His instillation-induced eye scratching behavior. saline instillation group (*n* = 5), 300 μg His (*n* = 6). **(A)** Number of scratches (Welch’s *t*-test: *P* < 0.05). **(B)** Scratching duration. **(C)** Time per scratching bout. **(D)** Latency. **(E–H)** CQ instillation-induced eye scratching behavior. saline instillation group (*n* = 5), 500 μg CQ (*n* = 6). **(E)** Number of scratches (Welch’s *t*-test: *P* < 0.05). **(F)** Scratching duration (Welch’s *t*-test: *P* < 0.01). **(G)** Time per scratching bout. **(H)** Latency. **(I–N)** Representative β-gal for labeling GRPR-expressing neurons and c-Fos immunostaining in the Sp5C dorsal horn of the Rosa26 loxP-stop-loxP-nlsLacZ (RNZ)-GRPR-Cre mice exposed to saline **(I, J)** or His **(K, L)** or CQ **(M, N)** instillation. **(J, L, N)** are enlargements of the boxed areas in panels **(I, K, M)**, respectively. Arrow head, double positive neurons for β-gal and c-Fos **(L, N)**. Bars = 100 μm (M); 10 μm (N). **(O–S)** Histological analysis with saline and His instillation. **(O)** The number of the β-gal positive GRPR neurons. **(P)** The number of c-Fos positive neurons (Mann–Whitney *U* test: *P* < 0.01). **(Q)** The number of β-gal and c-Fos double positive neurons. **(R)** The percentage of double positive neurons in the total c-Fos-expressing neurons. **(S)** The percentage of double positive neurons in the total GRPR-expressing neurons (Mann–Whitney *U* test: *P* < 0.05). **(T–Y)** Histological analysis with saline and CQ instillation. **(T)** The number of the β-gal positive GRPR neurons. **(U)** The number of c-Fos positive neurons (Mann–Whitney *U* test: *P* < 0.05). **(W)** The number of β-gal and c-Fos double positive neurons (Mann–Whitney *U* test: *P* < 0.01). **(X)** The percentage of double positive neurons in the total c-Fos-expressing neurons. (Mann–Whitney *U* test: *P* < 0.05). **(Y)** The percentage of double positive neurons in the total GRPR-expressing neurons (Mann–Whitney *U* test: *P* < 0.01). Saline instillation group (*n* = 5), 300 μg His (*n* = 6), 500 μg CQ (*n* = 5).

Since itchy eye stimulation increased the c-Fos expression in the Sp5C, we subsequently studied RNZ-Grpr-Cre mice in which GRPR-expressing neurons were labeled with β-gal to examine the neural activity in the GRPR-expressing neurons of the Sp5C. First, we examined the His and CQ induced eye scratching behavior of RNZ-Grpr-Cre female mice (Figures 3A–H). High dose 300 μg His instillation (*n* = 6) significantly increased the number of scratches compared to the saline instillation group (*n* = 5) (Welch’s *t*-test; *P < 0.05, *P* = 0.048) (Figure 3A). High dose 500 μg CQ instillation (*n* = 6) showed a significant increase the number of scratches (Welch’s *t*-test: *P < 0.05, *P* = 0.017) (Figure 3E) and scratching duration (Welch’s *t*-test: **P < 0.01, *P* = 0.007) (Figure 3F) compared to the control group (*n* = 5). Next, the neuronal activity of GRPR-expressing neurons in the Sp5C during itch stimulation of the eyes was examined by immunofluorescence using female RNZ-Grpr-Cre mice (Figures 3I–N). GRPR-expressing neurons labeled with β-gal were observed throughout the dorsal horns of the Sp5C (Figures 3I–N). The number of GRPR-expressing neurons labeled with β-gal in the Sp5C did not significantly vary in 300 μg His (*n* = 6) or 500 μg CQ (*n* = 5) instillation group compared to the saline group (*n* = 5), (Figures 3O, T). His or CQ instillation significantly increased the number of c-Fos expression in the Sp5C compared to the control group (Mann–Whitney *U* test: **P < 0.01, *P* = 0. 009, saline vs. His; *P < 0.05, *P* = 0.016, saline vs. CQ) (Figures 3P, U), and the number of double-positive neurons for GRPR and c-Fos was significantly higher in the CQ instillation group than in the control group (Mann–Whitney *U* test: *P* = 0.052, saline vs. His; **P < 0.01, *P* = 0.008, saline vs. CQ) (Figures 3Q, W). CQ instillation resulted in a significantly higher percentage of double-positive neurons among the total c-Fos-expressing neurons in the Sp5C than in the control group (Mann–Whitney *U* test: *P < 0.05, *P* = 0.032, saline vs. CQ) (Figure 3X). His and CQ instillation resulted in a significantly higher percentage of double-positive neurons among the total GRPR-expressing neurons in the Sp5C than in the control group (Mann–Whitney *U* test: *P < 0.05, *P* = 0.03, saline vs. His; **P < 0.01, *P* = 0.008, saline vs. CQ) (Figures 3S, Y). These results showed that both the His and CQ stimuli in the eyes increased the neural activity of the Sp5C neurons and GRPR-expressing neurons in the Sp5C.

### 3.4 Intracisternal administration of the GRPR agonist, NMC induced excessive facial scratching behavior

**FIGURE 4:**
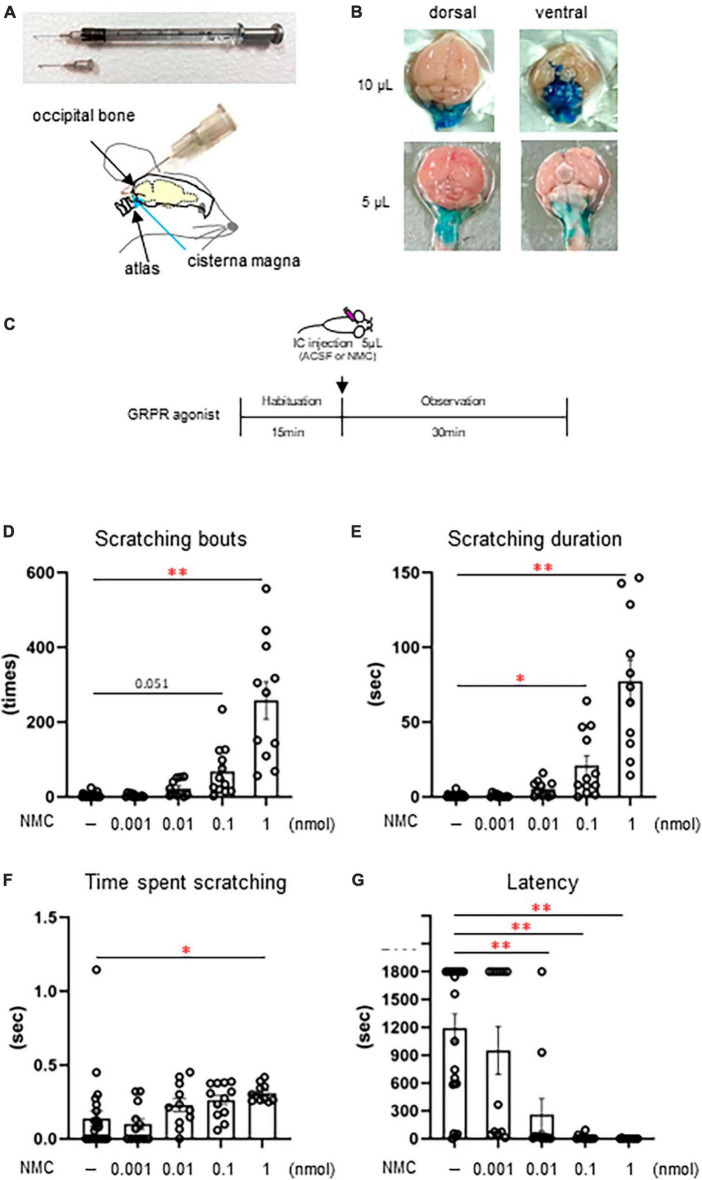
Intracisternal administration of gastrin-releasing peptide receptor (GRPR) agonist, neuromedin C (NMC) induced excessive facial scratching behavior **(A)** Schematic showing the intracisternal injection. **(B)** Diffusion of 1% of methylene blue by intracisternal administration. **(C)** Time course. **(D–G)** Facial scratching behavior. **(D)** Number of scratches (*post-hoc* Dunnett test: *P* < 0.01, artificial cerebrospinal fluid [ACSF] vs. 1 nmol NMC). **(E)** Scratching duration (*post-hoc* Dunnett test: *P* < 0.05, ACSF vs. 0.1 nmol, *P* < 0.01, ACSF vs. 1 nmol NMC). **(F)** Time per scratching bout (*post-hoc* Dunnett test: *P* < 0.05, ACSF vs. 1 nmol NMC). **(G)** Latency (*post-hoc* Dunnett test: *P* < 0.01, ACSF vs. 0.01 nmol or 0.1 nmol or 1 nmol NMC). ACSF-treated group (*n* = 23), 0.001 nmol NMC (*n* = 12), 0.01 nmol NMC (*n* = 11), 0.1 nmol NMC (*n* = 12), 1 nmol NMC (*n* = 11). IC, intracisternal injection.

Since the histological analysis revealed the activation of GRPR-expressing Sp5C neurons by itchy eye stimulation, we next examined the conditions of intracisternal administration in female mice (Figure 4A) to directly activate the GRPR of Sp5C. After examining the diffusion site by dye administration, we selected a 5 μL volume because 10 μL of intracisternal administration spread widely over the ventral and dorsal parts of the brainstem (Figure 4B), whereas 5 μL showed localized dye deposition around the Sp5C (Figure 4B). Intracisternal administration of GRPR agonist NMC (Figure 4C) induced excessive facial and head scratching behavior including hindfoot facial scratching, fine and fast forefeet movements, and facial wiping behavior of both the forefeet in a dose-dependent manner (ACSF: *n* = 23; 0.001 nmol NMC: *n* = 12; 0.01 nmol NMC: *n* = 11; 0.1 nmol NMC: *n* = 12; 1 nmol NMC; *n* = 11) (Figures 4D–G) (Supplementary Material: Movie 5). Lowest dose 0.001 nmol NMC showed no difference in the scratching behavior from the control ACSF (Figures 4D–G). Intracisternal injection of 1 nmol NMC significantly increased the number of scratches [*F*(4, 64) = 26.918, *P* < 0.001, *post-hoc* Dunnett’s test: *P* = 0.051, ACSF vs. 0.1 nmol NMC, **P < 0.001, ACSF vs. 1 nmol NMC] (Figure 4D). Intracisternal injection of 0.1 nmol or 1 nmol NMC significantly increased the scratching duration [*F*(4, 64) = 29.348, *P* < 0.001, *post-hoc* Dunnett’s test: *P < 0.05, *P* = 0.030, ACSF vs. 0.1 nmol NMC, **P < 0.001, ACSF vs. 1 nmol NMC] (Figure 4E). Intracisternal injection of 1 nmol NMC significantly increased the time per scratching bout [*F*(4, 64) = 3.214, *P* = 0.018, *post-hoc* Dunnett’s test: *P < 0.05, *P* = 0.035, ACSF vs. 1 nmol NMC] (Figure 4F) Intracisternal injection of 0.01 nmol, 0.1 nmol, or 1 nmol NMC significantly decreased the latency [*F*(4, 64) = 12.835, *P* < 0.001, *post-hoc* Dunnett’s test: **P < 0.001, ACSF vs. 0.01 nmol NMC, 0.1 nmol NMC, or 1 nmol NMC] (Figure 4G). These results indicated that the activation of GRPR in the Sp5C causes facial itch, even in the absence of itch stimuli on the face.

### 3.5 Effect of intracisternal administration of GRPR antagonist on eye scratching behavior evoked by histaminergic and non-histaminergic pruritogens

**FIGURE 5:**
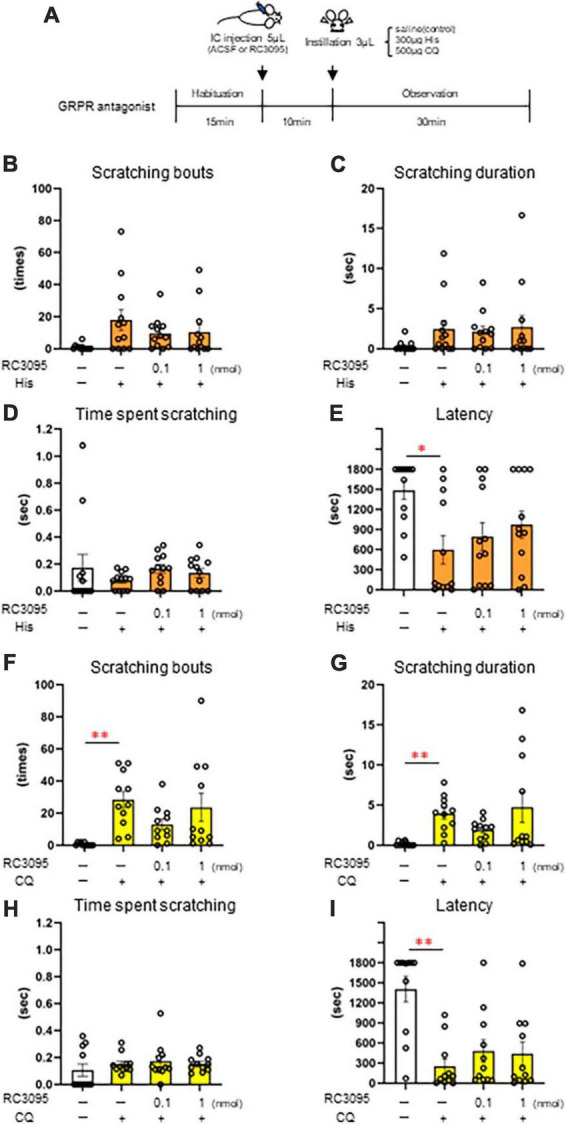
Effect of intracisternal administration of gastrin-releasing peptide receptor (GRPR) antagonist, RC-3095 on eye scratching behavior evoked by histamine (His) or chloroquine (CQ). **(A)** Time course. **(B–E)** 300 μg His instillation with artificial cerebrospinal fluid (ACSF) or RC-3095 intracisternal injection. **(B)** Number of scratches. **(C)** Scratching duration. **(D)** Time per scratching bout. **(F)** His instillation with ACSF showed the short latency than saline instillation with ACSF (*post-hoc* Durbin-Conover test: *P* < 0.05). **(F–I)** 500 μg CQ instillation with ACSF or RC-3095 intracisternal injection. **(F,G)** CQ instillation with ACSF increased the scratching number and scratching duration than saline instillation with ACSF (*post-hoc* Durbin-Conover test: *P* < 0.01). **(H)** Time per scratching bout. **(I)** CQ instillation with ACSF showed the short latency than saline instillation with ACSF (*post-hoc* Durbin-Conover test: *P* < 0.01). His instillation group (*n* = 12), CQ instillation (*n* = 11). IC, intracisternal injection.

Since intracisternal administration of the GRPR agonist induces excessive facial scratching, we investigated the effect of the GRPR antagonist on itchy eyes in female mice. To inhibit the GRPR function in the Sp5C, 10 min after the intracisternal administration of the 0.1 nmol or 1 nmol GRPR antagonist RC-3095 or ACSF as a control, saline, 300 μg His (*n* = 12) (Figures 5B–E), or 500 μg CQ (*n* = 11) (Figures 5F–I) was instilled into the eyes (Figure 5A). GRPR antagonist did not suppress His-induced eye scratching number [χ^2^(3) = 6.13, *P* = 0.105] (Figure 5B), scratching duration [χ^2^(3) = 6.39, *P* = 0.094] (Figure 5C), time per scratching bout [χ^2^(3) = 5.24, *P* = 0.155] (Figure 5D), and latency [χ^2^(3) = 8.26, *P* = 0.041, *post-hoc* Durbin-Conover: *P < 0.05, *P* = 0.03, ACSF/saline vs. ACSF/300 μg His] (Figure 5E). GRPR antagonist also did not suppress CQ-induced eye scratching number [χ^2^(3) = 11.5, *P* = 0.009, *post-hoc* Durbin-Conover: **P < 0.01, *P* = 0.006, ACSF/saline vs. ACSF/500 μg CQ] (Figure 5F), scratching duration [χ^2^(3) = 12.7, *P* = 0.005, *post-hoc* Durbin-Conover: **P < 0.01, *P* = 0.006, ACSF/saline vs. ACSF/500 μg CQ] (Figure 5G), time per scratching bout [χ^2^(3) = 2.39, *P* = 0.495] (Figure 5H), and latency [χ^2^(3) = 12.7, *P* = 0.005, *post-hoc* Durbin-Conover: **P < 0.01, *P* = 0.006, ACSF/saline vs. ACSF/500 μg CQ] (Figure 5I). In conclusion, intracisternal administration of a 0.1 nmol or 1 nmol RC-3095 did not significantly suppress His- or CQ-induced eye scratching behavior.

### 3.6 Effect of toxin-induced depletion of the GRPR-expressing neurons on eye scratching behavior evoked by histaminergic and non-histaminergic pruritogens

**FIGURE 6:**
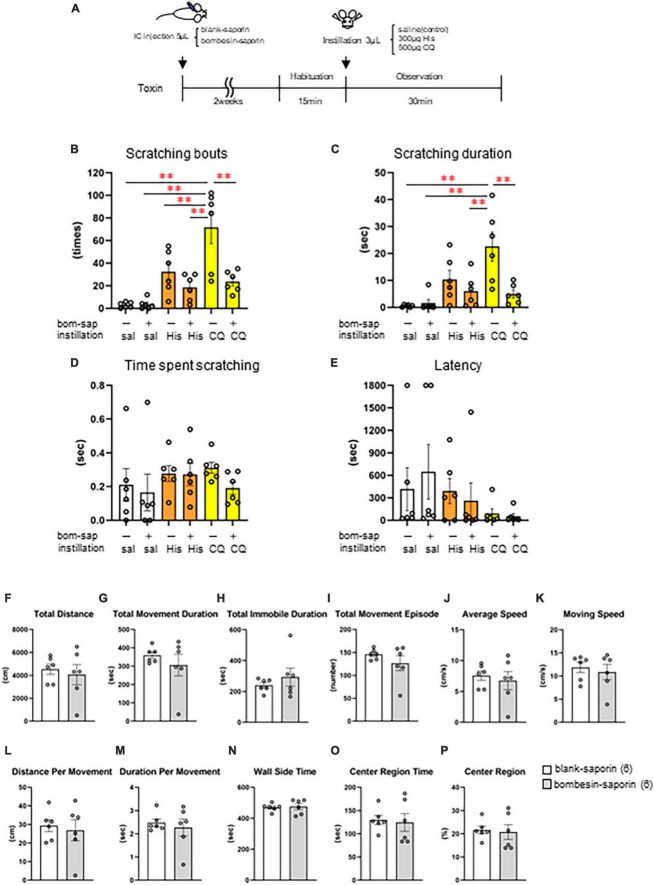
Effect of toxin-induced depletion of gastrin-releasing peptide receptor (GRPR)-expressing neurons on eye scratching behavior evoked by histamine (His) or chloroquine (CQ). **(A)** Time course. **(B)** Bombesin-saporin-treated group with CQ instillation suppressed the number of scratches compared to blank-saporin with CQ group (*post-hoc* Tukey test: *P* < 0.01). **(C)** Bombesin-saporin-treated group with CQ instillation suppressed scratching duration compared to blank-saporin with CQ group (*post-hoc* Tukey test: *P* < 0.01). **(D)** Time per scratching bout. **(E)** Latency. **(F–P)** Open field test. **(F)** Total distance. **(G)** Total movement duration. **(H)** Total immobile duration. **(I)** Total movement episode. **(J)** Average speed. **(K)** Moving speed. **(L)** Distance per movement. **(M)** Duration per movement. **(N)** Wall side time. **(O)** Center region time. **(P)** Center region (%). There were no differences in any of the parameters between the two groups (Mann–Whitney *U* test: *P* > 0.05). Blank-saporin-treated group (*n* = 6), Bombesin-saporin-treated group (*n* = 6). IC, intracisternal injection.

Finally, we induced cell death in the GRPR-expressing neurons in the medulla using toxin and examined whether His- or CQ-induced eye itch transmission was altered. Eye scratching behavior was analyzed by intracisternal administration of blank-saporin (*n* = 6) or bombesin-saporin (*n* = 6) that binds with high affinity to GRPR 2 weeks before the behavioral experiment, followed by saline, 300 μg His, or 500 μg CQ instillation in female mice (Figure 6A). There were differences in the pattern of the number of scratches and the scratching duration among groups [scratching number: *F*(5, 30) = 12.5, *P* < 0.001, scratching duration: *F*(5, 30) = 7.71, *P* < 0.001, the time per scratching bout: *F*(5, 30) = 0.652, *P* = 0.662, latency: *F*(5, 30) = 0.975, *P* = 0.449]. Bombesin-saporin did not suppress His-induced eye scratching behavior (Figures 6B–E), but suppressed CQ-induced eye scratching number (*post-hoc* Tukey test: **P < 0.01; blank-saporin/CQ vs. bombesin-saporin/CQ) and scratching duration (*post-hoc* Tukey test: **P < 0.01; blank-saporin/CQ vs. bombesin-saporin/CQ). These results suggest that the GRPR-expressing neurons in Sp5C are important for the transmission of CQ-induced itchy eyes. Since the GRPR is also involved in motor function, we performed an open field test to examine whether the CQ-induced scratching behavior reduced by bombesin-saporin was associated with reduced motor function in the GRPR. No differences were observed between the blank- and bombesin-saporin groups in each parameter reflecting motor function (e.g., total distance, total movement duration, total movement episode, average speed, and moving speed) and anxiety (e.g., total distance, total immobile duration, wall-side time, and center region time) (Mann–Whitney *U* test: *P* > 0.05) (Figures 6F–P).

## 4 Discussion

### 4.1 Eye scratching behavior induced by histaminergic and non-histaminergic pruritogens in male and female mice

In the present study, we examined the effect of His as a histaminergic and CQ as a non-histaminergic pruritogen on eye-scratching behavior in mice. We observed His and CQ-induced hindfoot scratching, fine and fast forefeet movements, and wiping with both forefeet in the eyes, which could be distinguished from grooming in mice, similar to previous reports (Nakano et al., 2009). Rats and guinea pigs also reportedly exhibit uninterrupted clusters of rapid forefeet movement behavior and hind-limb scratching during eye itch stimulation (Minami and Kamei, 2004). Thus, rodents exhibit scratching behavior with their forefeet and hindfoot when they receive an itch stimulus in the eyes, which would indicate an attempt to remove the itching substance. More detailed analysis is needed to determine the proportion of forelimb versus hindlimb scratching behavior by type and concentration of the pruritogens. In addition, an increase in scratching behavior was accompanied by an increase in whole-body grooming behavior. Since grooming is considered to have a wide variety of components, such as behavior associated with itching, behavior to get rid of foreign substances, behavior induced by anxiety, and behavior to calm oneself, further analysis is required to understand the relationship between grooming and itch in the future.

We found that female mice demonstrated a higher scratching duration than males at high concentrations of His and higher scratching behavior in a concentration-dependent manner for both His and CQ. The time per scratching bout at high concentrations of His and CQ also demonstrated significant sex differences in females, indicating the variation in itch sensitivity and coping strategies between males and females. Fujii et al. (2005, 2009) reported that mice with atopic-dermatitis-like skin lesions induced by special diet showed normal scratching frequency and cumulative scratching duration but prolonged the time per scratching bout. Scratching includes a series of behaviors from recognizing itch stimulus, to initiating and to converging the scratching behavior, and involves the perception of itch intensity, emotional cognition, and reward system. Therefore, not only the number of scratches, but also the scratching duration or time per scratching bout are important in the analysis of itching. Significant female sex differences in allergic conjunctivitis have been reported according to the region (Takamura et al., 2010, 2017; Hong et al., 2016; Miyazaki et al., 2020). The reason for sex differences in the prevalence of allergic conjunctivitis is unclear; however, sex steroid hormones may be involved. Although the transmission of eye itching is innervated by the trigeminal sensory system and not by the spinal sensory system, we demonstrated that itch increases in response to 17β-estradiol administration following castration in female rats, and that the spinal GRP/GRPR system is involved (Takanami et al., 2021). It has also been reported that the ablation of estrogen receptor α expressing interneurons in the spinal cord reduces chemically induced modalities of pain and itch, and pruritogen-induced scratching in both male and female mice (Tran et al., 2020). Furthermore, in the periaqueductal gray, which is part of the descending inhibitory pathway, estrogen and androgen receptors are expressed in both males and females. However, sex-related differences exist in their expression and distribution (Loyd and Murphy, 2008). Thus, since sex steroid hormones are widely distributed in the central nervous system, involved in the transmission of sensation, and expressed in the peripheral tissues, sex steroid hormone receptors may influence the sensitivity of perception. Although C57BL/6J mice were used in this study, further analysis is needed to determine the presence of sex differences based on sensitivity to itchy eyes, as species and strain differences in perception have been reported.

### 4.2 Itchy eyes stimuli activated the GRPR-expressing neurons in the Sp5C

The present histological analysis demonstrated that the itch stimulus to the eye activated neurons in the superficial layers of Sp5C. This c-Fos expression suggests that neurons were activated by physical stimulation due to scratching behavior, in addition to the itch induced by pruritogens. The trigeminal nerve is divided into three main branches, the ophthalmic (V1), maxillary (V2), and mandibular (V3) nerves. Conjunctival sensations are innervated by the ophthalmic (V1) and maxillary (V2) nerves. The V1, V2, and V3 nerves input ventral, lateral, and dorsal to the Sp5C. In the present study, GRPR-expressing cells were found throughout the Sp5C. In contrast, c-Fos, which is upregulated in itchy eyes, is mainly localized in the input area of the V1 and V2 nerves. His- and CQ-induced itchy eyes increased the number of GRPR and c-Fos double-positive neurons in the Sp5C, similar to the results reported for His-induced itchy eyes in rats (Katayama et al., 2022). These results indicated that eye stimulation with His and CQ activated the GRPR-expressing neurons in the Sp5C in mice.

### 4.3 Effect of intracisternal administration of GRPR antagonist on eye scratching behavior

Since the histological analysis demonstrated that eye irritation by His or CQ activated GRPR-expressing neurons of Sp5C, we predicted that inhibition of the GRPR of the Sp5C would suppress itchy eyes. However, intracisternal administration of a GRPR antagonist did not suppress His- or CQ-induced eye-scratching behavior. Intrathecal injection of a GRPR antagonist targeting the spinal sensory system in mice reportedly significantly suppressed non-histaminergic pruritogen-induced scratching behavior, but not that induced by a histaminergic pruritogen (Sun and Chen, 2007; Akiyama et al., 2014; Kiguchi et al., 2020). The intracisternal administration of a GRPR antagonist in our study showed a tendency to decrease CQ-induced scratching, similar to that of previous reports in the spinal somatosensory system, but did not reduce His-induced scratching (Sun and Chen, 2007; Akiyama et al., 2014; Kiguchi et al., 2020). These results indicate that itchy eyes induced by non-histaminergic substances may be transmitted to the brain via the GRPR in the medulla oblongata. Recently, Huang et al. (2018) discovered the neural basis underlying the dichotomy between ocular itch and pain and indicated that neuromedin B (NMB) and NMB receptor signaling in the trigeminal sensory system is important for conjunctival itch transmission. NMB receptor neurons function upstream of the GRPR neurons during itch transmission in the spinal somatosensory system of mice (Zhao et al., 2014; Wan et al., 2017). Considering these findings and the results of the present histological analysis, GRPR-expressing neurons may receive itchy eye signals mediated by the NMB/NMB receptors in the Sp5C.

### 4.4 Effect of intracisternal administration of bombesin-saporin on eye scratching behavior

Previous studies targeting the spinal sensory system in mice have shown that the intrathecal injection of 400 ng bombesin-saporin significantly reduced the number of spinal GRPR-expressing neurons and suppressed scratching behavior induced by histaminergic pruritogens (His, compound 48/80, 5-HT, endothelin-1), non-histaminergic pruritogens (protease-activated receptor-2, CQ), and chronic itch models (Sun et al., 2009). Genetically modified rats, in which GRPR-expressing neurons in the Sp5C were largely abolished using the toxin receptor-mediated cell knockout method by administering diphtheria toxin directly into the brain, demonstrated significantly suppressed His-induced eye scratching behavior (Katayama et al., 2022). We performed a behavioral analysis of itchy eyes 2 weeks after intracisternal administration of 500 ng bombesin-saporin to induce the loss of GRPR-expressing neurons in the medulla oblongata, as reported by Sun et al. (2009). In this study, bombesin-saporin treatment significantly suppressed CQ-induced eye-scratching behavior, indicating that GRPR-expressing neurons in the Sp5C are important for transmitting CQ-induced eye-itch stimuli. In contrast, the GRPR-expressing neurons were not directly involved in the transmission of His-induced itchy eyes. Considering that our histological analysis demonstrated that both His and CQ instillation significantly increased the neural activity of the GRPR-expressing neurons in the Sp5C, it is possible that His-induced itch is indirectly involved in the activity of GRPR-expressing neurons. Previous studies indicated that NMBR-expressing neurons in the spinal cord are required for itch, including histaminergic itch (Wan et al., 2017). Considering these studies, it is possible that His-induced itch is transmitted through the NMBR-expressing neurons, which is upstream of the GRPR also in trigeminal sensory system. Considering other possibilities, the limited diffusion of the toxin following intracisternal injection could be one of the reasons why bombesin-saporin did not suppress His-induced eye-scratching behavior in this study. Future analyses using direct toxin administration, chemogenetics, or optogenetics are required to suppress or ablate Sp5C GRPR- or GRPR-expressing neurons more specifically. In summary, GRPR-expressing neurons in the Sp5C may be important for non-histaminergic CQ-mediated eye itch transmission.

### 4.5 Intracisternal administration of GRPR agonist induced excessive facial scratching behavior

In the present study, the intracisternal administration of a GRPR agonist, NMC induced excessive facial scratching in a dose-dependent manner. This indicates that NMC binding to the GRPR in the Sp5C induced facial itching even in the absence of a direct itch stimulus input to the face; thus, brain stimulation could induce itching in the face. This result is similar to that of a previous study targeting the spinal sensory system in mice, in which intrathecal injection of GRP induced scratching behavior (Sun and Chen, 2007; Sukhtankar and Ko, 2013). NMC is the C-terminus of the bioactive decapeptides of GRP, and the NMC sequence is the binding site of GRPR (Peng et al., 2023) and has demonstrated various bioactivities (Ladenheim et al., 1996; Sakamoto et al., 2008). NMC was used in this study because its small molecular allows it to easily penetrate tissues and is suitable for *in vivo* analysis. Furthermore, evolutionarily, NMC sequences are conserved across vertebrates (Takanami et al., 2022), suggesting that NMC play an important biological function. Considered together, our results indicate that GRPR in the trigeminal system is also strongly involved in itch transmission in the facial area.

In the present study, we analyzed the involvement of GRP/GRPR in the transmission of acute itchy eyes and did not analyze the effect of GRP/GRPR on pain in the trigeminal system or in pathological models of allergic conjunctivitis. The elucidation of these functions is important for future research. In summary, our study demonstrated for the first time that GRPR-expressing neurons localized in the Sp5C are involved in the non-histaminergic itch in the trigeminal sensory system. Furthermore, elucidating histaminergic and non-histaminergic eye itch transmission is expected to lead to better understanding and treatment of itch caused by conjunctivitis.

## Data availability statement

The raw data supporting the conclusions of this article will be made available by the authors, without undue reservation.

## Ethics statement

The animal study was approved by the National Institute of Genetics and Nara Women’s University Committee for Animal Care and Use. The study was conducted in accordance with the local legislation and institutional requirements.

## Author contributions

AO: Formal analysis, Investigation, Visualization, Writing – review and editing. MH: Formal analysis, Visualization, Writing – review and editing. YS: Investigation, Writing – review and editing. HS: Funding acquisition, Investigation, Resources, Writing – review and editing. TK: Funding acquisition, Investigation, Resources, Writing – review and editing. KT: Conceptualization, Formal analysis, Funding acquisition, Investigation, Supervision, Writing – original draft, Validation, Visualization, Writing – review and editing. MK: Formal analysis, Investigation, Validation, Writing – review and editing. RI: Formal analysis, Investigation, Validation, Visualization, Writing – review and editing. YI: Formal analysis, Investigation, Writing – review and editing, Validation.

## Footnotes

1
https://www.jamovi.org

## Supplementary material

Supplementary Movie 1 Hindfoot eye scratching.

Supplementary Movie 2 Fine and fast forefeet movements.

Supplementary Movie 3 Wiping with both forefeet.

Supplementary Movie 4 Grooming without eye stimulation.

Supplementary Movie 5 Facial and head itch behaviors.

Supplementary Movie 1–3 500 μg CQ instillation into the eyes.

Supplementary Movie 4 Spontaneous.

Supplementary Movie 5 Intracisternal administration of 1 nmol NMC.
